# Identical probes on different high-density oligonucleotide microarrays can produce different measurements of gene expression

**DOI:** 10.1186/1471-2164-7-153

**Published:** 2006-06-15

**Authors:** LanMin Zhang, Sean J Yoder, Steven A Enkemann

**Affiliations:** 1 Microarray Core Laboratory, H. Lee Moffitt Cancer Center and Research Institute, SRB2, 12902 Magnolia Drive, Tampa, Florida 33612, USA

## Abstract

**Background:**

There are many potential sources of variability in a microarray experiment. Variation can arise from many aspects of the collection and processing of samples for gene expression analysis. Oligonucleotide-based arrays are thought to minimize one source of variability as identical oligonucleotides are expected to recognize the same transcripts during hybridization.

**Results:**

We demonstrate that although the probes on the U133A GeneChip arrays are identical in sequence to probes designed for the U133 Plus 2.0 arrays the values obtained from an experimental hybridization can be quite different. Nearly half of the probesets in common between the two array types can produce slightly different values from the same sample. Nearly 70% of the individual probes in these probesets produced array specific differences.

**Conclusion:**

The context of the probe may also contribute some bias to the final measured value of gene expression. At a minimum, this should add an extra level of caution when considering the direct comparison of experiments performed in two microarray formats. More importantly, this suggests that it may not be possible to know which value is the most accurate representation of a biological sample when comparing two formats.

## Background

Microarrays are rapidly becoming a standard tool for molecular biologists. The technology can reveal a multitude of information from even the simplest biological experiment. The technique is primarily used by scientists to generate ideas in experiments colloquially referred to as "fishing expeditions". However, the result of even simple experiments is hundreds to thousands of gene expression changes. Because of this the cost of validation is prohibitive. The microarray technology itself is expensive and this often prevents replication. Furthermore, the preliminary experiments are usually composed of the minimum number of samples absolutely necessary to address the question. This minimalistic approach is also used for other common microarray experiments. Rather than replicate an experiment or increase the size of a study, it is increasingly common for researchers to turn to other published microarray experiments to attempt to verify or expand their findings. Early attempts at cross-platform verification of microarray experiments utilized data generated in other laboratories from independent experiments [1-6]. These attempts produced limited success and undoubtedly many more studies went unpublished because of the difficulty in reproducing the results of one assay in a second platform.

However, recent studies have shed a more positive light on cross-platform comparisons. Several studies have now shown that the probes used to detect transcripts are the root cause of differences between platforms [7-10]. The probes on some arrays do not detect the transcripts attributed to them [11,12]. This is especially a concern for cDNA-based arrays [13,14]. Many commercial platforms are utilizing oligonucleotide-based probes. However, commercial array producers do not always accurately identify the targets of their probes [15,16]. There are also limitations to our understanding of the transcriptome and therefore two different probes that, theoretically, detect the same transcript may produce different measurements in microarray experiments because of cross-hybridization from unknown splice variants, gene families, and transcribed pseudogenes. Nonetheless, the correlation between different arrays is quite good if one restricts the analysis to sequence matched probes [9,10,17-19].

Oligonucleotide based arrays appear to have higher resolution and lower variability than cDNA based arrays [2,20,21]. They are also easier to compare across-platforms because the sequences can be easily cross-matched. Comparison is even easier when the exact same sequence is used on two different arrays. The most recent Affymetrix GeneChips contain probes that were previously available on earlier arrays. Therefore the sequences are identical and would be expected to produce similar measurements from the exact same samples. We tested this assumption and found that identical probes on the two arrays can produce different measurements.

## Results

**Figure 1:**
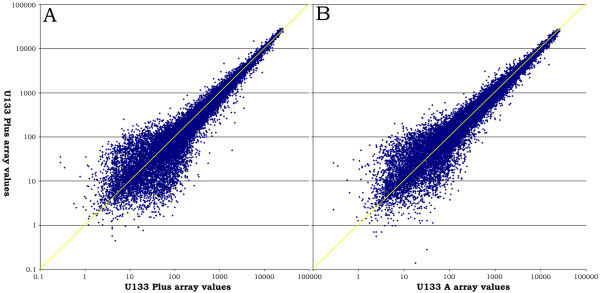
**Scatterplots of array data generated from independent samples of Universal Human Reference RNA**. The values plotted are derived from the Signal values calculated for the probesets found on both U133A arrays and U133 Plus arrays. A. Two samples of Reference RNA hybridized to U133 Plus 2.0 arrays are plotted on the X and Y axes. B The same sample hybridized to a U133 Plus 2.0 array plotted on the Y axis is now compared to the values obtained from a U133A array (X axis).

The Stratagene Universal Human Reference RNA standard is frequently used in microarray experiments to provide a standard reference for comparison purposes. We previously processed this RNA standard several times for microarray experiments using the U133A and U133B arrays from Affymetrix. When the newer U133 Plus 2.0 (U133 Plus) arrays were introduced we also processed this Universal Reference RNA on the new array. The U133 Plus arrays contain 54,675 probesets. This includes 22,277 probesets with identical probes to those used on the U133A arrays, 22,645 probesets from the U133B arrays, and an additional 9927 newly designed probesets. After processing the first few Reference RNA samples on the U133 Plus arrays we evaluated the similarity of these measurements to those acquired with the corresponding probesets on the U133A arrays. We directly compared the values obtained from 12 U133 Plus arrays to those obtained from 12 U133A arrays. We extracted the data from the probesets found on both array types and performed invariant set normalization to place these sets of data on the same scale [22,23]. Following this normalization we compared the U133A array values to the U133 Plus array values for each common probeset. We were surprised to find that 10,552 probesets gave a different value on the Plus chip than on the U133A chip (Student's t-test, p-value < 0.05). This is nearly half of the probesets shared by both arrays. Overall, the array data from any of the U133 Plus arrays looks very similar to array data from any of the U133A arrays (figure 1). The correlation between any two samples was very high (Maximum R = .9867, Minimum R = .8984, Average R = .9621). Yet, for individual probesets, the values appeared to fall into either a group comprised of U133A array values or a group comprised of U133 Plus array values. Scatterplots and correlation coefficients are frequently used to demonstrate the reproducibility of microarray data. However, this data demonstrates how misleading these and other gross measures of similarity can be for evaluating how different two sets of microarray data can be. The overall correlation was greater than .96 across 22,227 probesets and yet nearly half of these probesets yielded measurable differences between the A chip relative to the Plus chip.

### Controlled hybridization experiment

We decided to investigate this phenomenon further to be certain that the differences were not due to unknown bias introduced during processing of the samples. To minimize the possible sources of bias, we processed 5 aliquots of the Reference RNA in parallel using the same reagents. Following the production of labeled RNA we prepared enough hybridization mixture from each preparation to hybridize both a U133A array and a U133 Plus array at the same time. We further processed the samples in parallel to minimize staining and scanning issues. Following this carefully controlled experiment we examined the U133A probeset values relative to the same probesets on the U133 Plus arrays. This time the t-test indicated that more than 5000 probesets were yielding different values on the two array types (p-value < 0.05). Nearly 10% of the evaluated probesets were strongly separated into distinct groups (2191 probesets, p-value < 0.01). There were 1568 probesets in which every recorded measurement from the A chip was larger than every recorded measurement from the Plus chip. There were 869 probesets in which every recorded measurement from the Plus chip was larger than every recorded measurement from the A chip. By chance alone we would only expect 43 such occurrences.

Although most microarray laboratories cannot mix array types for batch methods such as RMA or MBEI, we were able to generate artificial A CEL files from the appropriate probe values on the U133 Plus CEL files. This allowed us to perform RMA on the 10 arrays. Following quantile normalization and the model-based signal estimation of RMA more than 9000 probesets produced different values indicating that the array specific differences are not a consequence of a specific method of normalization or signal estimation. In addition, the A array files fabricated from the Plus array data allowed us to perform several types of normalization at the probe or probeset level similar to the detailed analysis of Choe *et al*. ([24]). None of the standard methods used for normalizing microarray data or calculating the probeset values reduced the number of differences between the two array formats to a level expected by chance (data not shown).

### Across array type versus within array type comparisons

**Figure 2:**
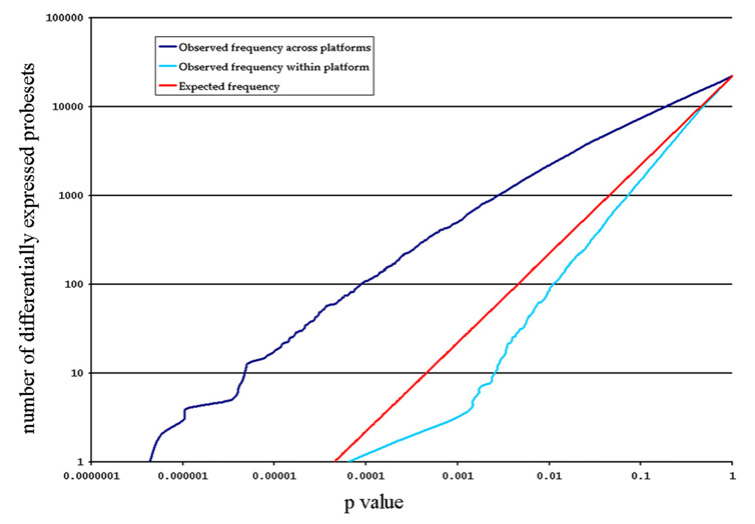
**Frequency of differential expression values observed as a function of p-value**. Plotted is the number of probesets at or below the indicated p-value for differential measurement when two pools of 5 arrays are compared. The p-values were calculated based on a standard t-test using one sampling of all the possible 5 by 5 comparisons.

We evaluated reference RNA samples hybridized to only the U133 Plus arrays. The 5 arrays hybridized above, coupled with the previous 12 arrays, yields a pool of 17 arrays. When we compared any random group of 5 arrays from this pool to a second random group of 5 arrays, we found ~500 probesets appeared to be different on average (t-test, p-value < 0.05). This is less than would be expected by chance if all the probes were producing random measurements. This is not surprising. RNA in the hybridized samples should bind to the appropriate probes providing directed signal. This reduces the number of probes producing random signal and therefore reduces the number of probesets producing divergent measurements when two groups of 5 are compared. This phenomenon occurs at all p-values (figure 2). The observed frequency of differential measurements is lower than the expected frequency when replicate arrays were taken from the same array type. In contrast, when the reference RNA was hybridized to different arrays types, more probesets showed differential expression than expected (figure 2). The number of differential measurements is much greater than expected by chance and far greater than expected for arrays hybridized with the exact same sample. This illustrates the prominent effect of array type on the measured value.

When arrays of the same type were compared the probesets that appeared to be differentially measured were probably all due to random noise. Approximately 500 probesets appeared to have group specific values in any 5 array to 5 array comparison (p-value < 0.05) within the same array type. A different 5 array by 5 array comparison yielded a different group of 500 probesets. Less than 2% of the probesets were in common between any two gene lists produced by such 5 by 5 comparisons. This illustrates that the apparent differences were nothing more than random variation that happened to fall into distinct distributions when the groups were formed. In contrast, the probesets found to be different when comparisons were made across the array types were more consistent. Approximately 5000 differences were observed at a p-value < 0.05 when 5 U133 Plus arrays were compared to a group of 5 U133A arrays. There was 60 to 85% overlap between any two lists generated by comparisons across array types. This suggests that the same probesets were being identified no matter which set of samples were chosen for comparison. This is strong evidence that the differences are real and not chance events.

**Figure 3:**
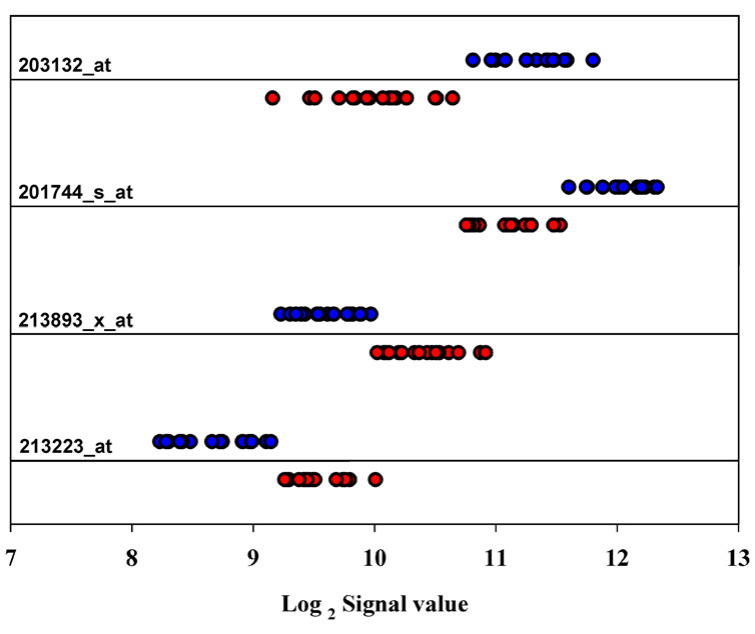
**Distribution of values obtained for selected probesets with U133A arrays and U133 Plus 2.0 arrays**. The probeset numbers are indicated on the left. Above each line and plotted with blue circles are the values observed with U133 Plus arrays. Below each line and plotted with red circles are the values observed for the corresponding probesets on the U133A arrays. Plotted are the log base 2 equivalents of the Signal values from 17 different arrays.

**Figure 4:**
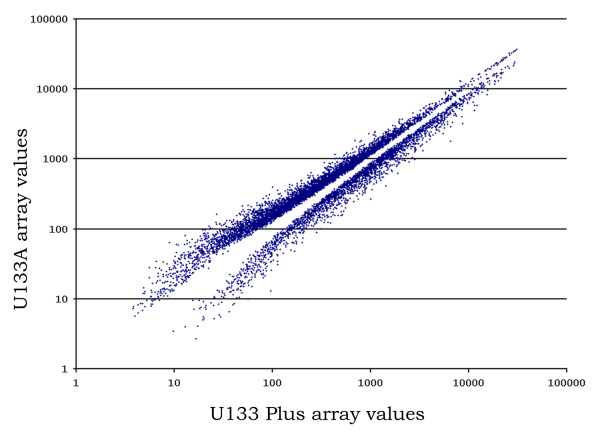
**Average measurement of the Universal Reference RNA on the U133A array relative to the U133 Plus array for those probesets exhibiting different measurements on the two arrays**. The probesets plotted were those in which the p-value was less than 0.01 when all 17 U133A arrays were compared to the 17 U133 Plus arrays. The average of the 17 arrays in one group is plotted against the average of the 17 arrays in the other group.

We ended up with a total of 34 replicate measures of the Universal Reference RNA, including 17 for each chip type. A comparison of the entire group of U133A arrays to the entire group of 17 U133 Plus arrays yielded more than 10,000 probesets with differential expression values specific to the array type. Therefore, as more data is added to the analysis the number of differential probesets increases rather than decreases, further suggesting that this is a function of the array and not due to chance. There were 101 probesets for which all 17 measurements for one array type were higher than all 17 measurements for the other array type. By chance this would be expected to occur only once in ~400,000 experiments of this type. We show 4 instances of this occurrence in Figure 3. This figure shows that sometimes the A chip-values were higher and sometimes the Plus chip-values were higher. It further illustrates that the affected probesets cover a range of hybridization values. All of the probesets depicted are well above the expression intensity observed for absent or weakly expressed transcripts. More than 2/3 of the probesets identified were not considered absent, by either Signal values thresholds or MAS 5.0 absent/present calls. To further demonstrate that this phenomenon is observed at all levels we plotted the average signal intensity of the U133A arrays against the average signal intensity obtained from the U133 Plus array for the probesets that showed a distinct distribution (p-value < 0.01, figure 4). This plot best illustrates that the phenomenon cannot be a consequence of normalization as one cannot correct a portion of these probesets without increasing the difference of another group.

### Latin square hybridization control

We have demonstrated that the measurements produced by the U133A arrays are different than the measurements from the U133 Plus arrays. At the same time the measurements are so similar that the correlation between array types is around 96%. In order to develop a frame of reference for the differences reported here we re-examined the latin-square data produced by Affymetrix scientists. This data set was produced from 42 hybridizations in which known quantities of RNA were added to a hybridization mixture containing labeled cRNA from HeLa cells. If we remove from consideration those probesets affected by the supplemented RNA, the rest of the data consists of 42 replicate measurements of the exact same hybridization mix. This is analogous to the experiment we performed by hybridizing the same cocktail to both a U133A chip and a U133 Plus chip. As a measure of variation we looked for probeset measurements that deviated more than 2-fold from the mean value of all the measurements. In both the latin square experiment and our experiments the probesets should have yielded the same value on all arrays as the same sample was measured. It is well known that the highest variation occurs for probesets with the lowest measurements. Therefore, we sliced the full data into three groups representing the highest expression values, the middle range, and the lowest measurements from the chips. Table 1 illustrates that hybridization, staining, and scanning related effects introduce some variation to the measurements obtained in a microarray experiment (column 1). More variation is observed if you also add the initial processing of the samples (column 2). In our experiments, where the same sample was hybridized to both array types, only the hybridization and post-hybridization steps were sources of variability. But rather than producing a similar degree of variation as the latin square data, more overall variation was observed than caused by the entire microarray process. (Compare column 3 to columns 1 and 2.)

### Evaluation of individual probes and probesets

The measurement differences between the A chip and the Plus chip are probeset specific but not transcript specific. This is illustrated by the results presented in table 2. The genes listed in table 2 are detected by more than one probeset on the arrays [16]. The first probeset of each group was identified because they produced extremely different distributions in the two chip types. The last column lists the p-value of the t-test as an indicator of the differences in the observed measurements. The preceding four columns list the maximum and minimum values recorded on the A chips and the maximum and minimum for the Plus chips. These columns provide a second view of how distinct the ranges can be and when they might overlap. The R value for the associated probesets is a measure of the correlation between the values obtained for that probeset and the first probeset of the group across 210 U133A arrays representing more than 70 different cell or tissue types. This is provided for reference to show that these probesets have a high degree of correlation with their cognate partners when only the U133A environment is considered. Yet their behavior across the array formats is distinctly different in many cases. For example, dimethyarginine dimethylaminohydrolase 2 and U2 small nuclear RNA auxiliary factor1-like 2 each have two probesets on the arrays that detects the same transcripts with similar intensity, but for one probeset the different arrays produce different values and for one probeset the two arrays produce overlapping values. Polyglutamine binding protein 1 and putative prostate cancer tumor suppressor also have two probesets detecting the same transcript, but in these cases one of the probesets generates higher values on the A chip and the other generates higher values on the Plus chip. Several other patterns are shown in table 3. Adducin 3 is detected by 4 probesets, each producing a different pattern when the A chip-values are compared to the Plus chip-values. These results further indicate that these differences are not the result of any bias introduced during processing or normalization, but must reside in individual probes on each respective array.

The transcript for KIAA0676 is detected by 4 different probesets. Three of these probesets behave very similarly on the two array types. The fourth probeset behaves in the opposite manner (table 2). The individual probes in each probeset, and their locations on the two arrays, are shown in table 3. The probesets that show the same behavior are nearly identical while the divergent probeset is distinctly different from the other three. On average, 9 of the 11 individual probes are identical for the 3 similarly acting probesets. The identically designed probes are also located side-by-side on the respective arrays. Therefore, these three probesets are virtually identical in both sequence and location on the arrays and identical in the bias seen between the array types. This fact was also true of several other parallel probesets we examined (data not shown).

We looked at the measurements recorded for the individual probes that comprise these probesets. We found that 10 of the 11 probes produced higher values on the U133A arrays than on the U133 Plus arrays for probesets 206431_x_at, 215994_x_at, and 212054_x_at. Conversely, the probeset 212052_s_at, which behaved in the opposite manner, had more individual probes yielding a higher value on the U133 Plus arrays. Therefore bias at the probe level appears to generate the bias observed for the full probeset. We looked at all the individual perfect match probes for the probesets that produced different measurements on the two array types. When the probeset produced a larger value in the U133A arrays an average of 7.6 individual probes (out of 11) were higher in the A arrays than in the U133 Plus arrays. Conversely, when the Plus chip produced a higher value, an average of 7.7 probes from these probesets were higher in the U133 Plus environment.

We suspected that this bias might be due to a little bleed over from the surrounding probes on the array. We examined a number of individual probes. In many cases brighter probes surrounded the probes producing the higher values; however this was not universally true. More consistently, it appeared that the probes yielding higher values were just a little brighter on the arrays with the higher measurements. This suggested a possible manufacturing difference as the underlying cause rather than bleed over from neighboring probesets.

## Discussion

One of the most important considerations in performing a microarray experiment is that the data obtained at the end is an accurate representation of the RNA present in the biomaterial used to start the process. Bias can lead to erroneous conclusions if the bias happens to track with the experimental condition evaluated or obscures the differences because the bias is larger than the biological effect. Bias can come from many sources [25-27]. When properly identified, bias can be corrected and a proper analysis can proceed. Our data demonstrates that the array itself can contribute bias to gene expression measurements. It is quite understandable how different probe sequences could lead to different measurements in gene expression arrays. However, our data shows that even identical probe sequences can yield slightly different measurements of gene expression.

The impact of probe level variation depends on the nature of the question addressed in a microarray experiment. For a simple experiment performed in either platform the observed performance of a gene should be substantially reproduced by the alternative platform. However, if clinical samples are being accumulated for a disease state, the differences between platforms might prevent the pooling of samples processed in both platforms. This depends on the differences one finds in the experimental system. Small differences might be obscured while large differences would still be observable. We observed that many of the probesets with target genes present in the Universal Reference RNA showed differences greater than 2-fold. This bias could compromise experiments where the number of samples evaluated might otherwise allow one to detect differences less than 2-fold.

Our data shows that microarray data can be very consistent at the same time as it shows a bias. Overall, any two arrays produced with the Universal Reference RNA yielded fairly consistent microarray results. The final measurements form a tight cluster of values for most probesets and the overall correlation was 0.96 for any two arrays. However, a significant number of probesets produced two distinctly different or partially overlapping distributions when the array formats were viewed separately (figure 2 and 3). Therefore the bias due to the array is clearly evident even though the overall correlation was very high. Sometimes a measurement was higher on the U133A arrays and sometimes it was higher in the U133 Plus arrays. This could even be true for the same transcript, as one probeset yielded higher values and another probeset, detecting the same transcript, yielded lower values than the alternative platform. This shows that the bias was not due to processing, hybridization, or normalization artifacts. This observation has larger implications. It illustrates that it may not be possible to have all probes on an array performing exactly the same way so that all transcripts could be measured on equivalent scales.

The trend in microarray research is towards generating larger sets of data for analyzing complex biological problems and diseases. Institutions are pooling resources to attain the large datasets required for analysis. Several groups have already evaluated the comparability of data produced at distant sites [28-30]. There are also attempts to directly compare gene expression data from quite dissimilar microarray platforms as well as serial analysis of gene expression (SAGE) and RT-PCR [31-34]. This push towards the direct comparison of microarray data from distant sites and dissimilar platforms is likely to continue. It is important to consider the differences in measurement observed on different array types.

Since their introduction, microarrays have been forecast for clinical use. Many people believe that patterns of gene expression will be used to identify disease states. Diagnosis, prognosis, and treatment options are all believed to lie in patterns of gene expression. While some people begin the search for these patterns, the microarray community has also been working towards improving the technology to produce more reliable results. Some sources of bias can be minimized or corrected. Others will simply be accepted as part of the process. Classifiers developed to interpret microarray data must allow for any variation that occurs in the measurement of individual probes. It remains to be determined whether the variation caused by the type of array will interfere with the performance or development of classifiers. For the present time it seems wise to use a single array type for the evaluation of microarray data. If the classification of samples relies on small gene expression differences, the classification may be microarray format specific. However, large differences will probably still be observed despite the small differences reported here between the U133A arrays and the U133 Plus arrays.

## Conclusion

Microarray data is often useful beyond the intentions of the original experiment. The microarray community is continuously developing standards for microarray processing and data management that allows scientists to utilize microarray data held in repositories. Much like sequence data, this stored gene expression information can be used in a multitude of creative ways. The comparison of microarray data between two formats, or even between two laboratories using the same format, requires knowledge about the sources of error that can arise in a microarray experiment. We have demonstrated that the same sequence can provide slightly different measurements of gene expression in different array formats. This implies that the comparison of microarray data between formats may require an additional, array specific, correction factor for each probe. The larger implication is that it may not be possible to establish equivalent correlations between the measured value in an array and the absolute value in a biological sample for every gene on an array. If sequence, as well as sequence context, introduces subtle adjustments to the final measured value of a transcript, then it may not be possible to know which measurement is the most accurate measurement of transcript abundance. Therefore attempts to perform cross-platform verification either have to use gene specific correction factors or be satisfied with similarity rather than exact replication.

## Methods

The source of RNA was the Universal Human Reference RNA from Stratagene (Catalog number 740000, Stratagene, La Jolla, CA) for all samples. This RNA was processed using the established Affymetrix protocols for the generation of biotin-labeled cRNA, hybidization, staining, and scanning. A more complete description of this process is available in the papers by Warrington *et al*. [35] or Dobbins *et al*. [28]. Hybridizations were performed as indicated in, either HG-U133A (U133A) arrays, or Human Genome U133 Plus 2.0 (U133 Plus) arrays from Affymetrix, Inc. (Santa Clara, CA). The U133A arrays were scanned on an Affymetrix GeneChip^® ^scanner 3000 at a 2.5 μm resolution and the U133 Plus chips were scanned at 1.56 μm resolution. All data was initially generated from the scanned image files using the MAS 5.0 software embedded in the GeneChip Operating Software from Affymetrix. The data was initially normalized globally to an average intensity of 500. The signal values from each array were then exported to a text file. The probesets in the U133 Plus arrays but not found on the U133A arrays were removed from these files and then all the arrays used in this study were renormalized in a single group using the iterative process described by Li and Wong as well as by Wang *et al*. [22,23]. This process insured that the final normalization was based on the most stable gene expression measurements across the arrays and that the probesets on the U133 Plus arrays, but not represented on the U133A arrays, did not influence the final normalized values.

All data used in this manuscript are publicly available for download. The initial samples placed on U133A arrays are available at the Gene Expression Database Portal ([36], experiment IDs 615–618). The samples hybridized to U133 Plus Arrays are from a series evaluating the effect of starting RNA quantity. The array data is available at the Gene Expression Omnibus ([37]) under accession number GSE3062. The samples processed and then hybridized to both a U133A array and a U133 Plus array are available under the accession number GSE3061.

The Latin Square data set used for comparison was generated by scientists at Affymetrix and is available from the company's website [38].

For the probe level analysis the values in each CEL file were loaded into a database. The probe values corresponding to those probesets found on the U133A arrays were extracted from each U133 Plus array and used to generate an artificial U133A CEL file. All the CEL files were then normalized to the same average value and individual probe values were compared across arrays. This technique also allowed comparisons using batch methods such as RMA or MBEI and perform other normalization techniques on the probe level data. The artificial U133A CEL files and corresponding U133A CEL files were loaded into a local implementation of the RMA program available through Bioconductor to calculate the expression values.

## Authors' contributions

LZ and SY produced the microarray data and some of the analysis presented in this manuscript. SE directed this project and wrote the majority of the manuscript.

## Figures and Tables

**Table 1:** Comparison between the variability introduced by hybridization and staining effects alone, the cDNA synthesis and transcription process, and cross-platform comparisons. Each value represents the percentage of probesets in each quantile of the array where measurements diverged more than 2-fold from the mean.

	**Single platform**	**Single platform**	**Cross platform**
	**Hybridization only**	**Total process**	**Hybridization only**

**Highest chip-values**	0.012%	0.27%	0.048%
**Middle chip-values**	0.88%	1.6%	1.31%
**Lowest chip-values**	6.4%	9.0%	13.0%
**Total**	7.3%	10.9%	14.1%

**Table 2:** Cross-platform behavior of selected probesets that detect the same transcript in the reference RNA.

**Transcript**	**Probe ID**	**R^a^**	**Plus chip range**		**A chip range**		**T-Test**
				
			**Max.**	**Min.**	**Max.**	**Min.**	
**Adducin 3 (gamma)**	201752_s_at	1	2317.2	1848.2	2714.6	2447.2	0.001
	201034_at	0.812	4713.7	3574	3949.1	3173.8	0.217
	201753_s_at	0.812	3723	2001.4	2015.5	1619.9	0.096
	205882_x_at	0.976	2054.1	1709.2	2275.5	1748.1	0.622
**dimethylarginine dimethylaminohydrolase 2**	202262_x_at	1	674	548.4	1040	745.2	0.001
	214909_s_at	0.889	744.8	428.4	707.6	543.3	0.994
**KIAA0676 protein**	206431_x_at	1	973	797.3	1329.1	1049.4	0.002
	212054_x_at	0.906	1115.7	749.5	1235.7	1052.3	0.041
	215994_x_at	0.925	1407.2	841.8	1570.2	1339.6	0.048
	212052_s_at	0.811	1685.6	1423.5	1397	1175.3	0.003
**Lysosomal-associated multi-spanning membrane protein-5**	201721_s_at	1	2944.9	2612	4151	3491.3	0.000
	201720_s_at	0.919	1768.6	1273.4	1531.3	1194.1	0.378
**nuclear transcription factor Y, gamma**	202216_x_at	1	792.9	586.3	1033.5	839.8	0.006
	211251_x_at	0.962	813.8	618.9	1070	885.3	0.001
	202215_s_at	0.639	861.6	319.3	798.2	512	0.887
	211797_s_at	0.761	672.2	428.9	864.9	541.3	0.237
**polyglutamine binding protein 1**	214527_s_at	1	1808.6	1378.3	2290.6	1868.9	0.006
	207769_s_at	0.907	1682.5	1505	1579	1280.6	0.02
**Putative prostate cancer tumor suppressor**	209228_x_at	1	1610.8	1375.8	2304.3	1875.1	0.001
	213423_x_at	0.916	2664.5	2295	2236.3	1600.9	0.033
**ribosomal protein, large, P0**	201033_x_at	1	53465.1	34223.5	67507	55973.4	0.014
	208856_x_at	0.967	56980.3	36192.7	63815.1	55584.1	0.136
	211720_x_at	0.927	53420.1	37636.2	66827.6	52346.1	0.021
	211972_x_at	0.792	50538.4	32930.5	56212.5	48139.1	0.051
**staufen, RNA binding protein (Drosophila)**	213037_x_at	1	6121.4	5809	6947.7	6309.3	0.002
	207320_x_at	0.945	6664.7	5179.8	7435.3	5183.5	0.5
	208948_s_at	0.854	5936.5	4774.2	5292.5	4762.6	0.256
**sulfotransferase (SULT1A3)**	209607_x_at	1	1798.6	1595.5	2015.2	1866	0.001
	210580_x_at	0.951	2497.1	1768.8	2435.6	2173.4	0.164
**U2 small nuclear RNA auxiliary factor 1-like 2**	208174_x_at	1	677.9	529	866.7	779.8	0.001
	213876_x_at	0.935	792.5	583.8	738.7	652.4	0.91

^a^R = the correlation coefficient between the 1^st ^probeset of each group and the indicated probeset across 210 U133A arrays representing more than 70 cell and tissue types.

**Table 3:** Sequence of the probes used to detect KIAA0676 protein and their location on the arrays.

	**Probe sequences for KIAA0676 protein**	**Plus chip location**		**A chip location**			**Probe sequences for KIAA0676 protein**	**Plus chip location**		**A chip location**		
	206431_x_at	X	Y	X	Y		215994_x_at	X	Y	X	Y	
1	ACACTTAGTCCTCCACAGTGGGTGG	360	191	527	107		CAAGTATGGGGCCCTGGCTGTGTTC	765	297	4	169	
2	AAGAGTGCAAGGTCTGCCAGGTCAG	669	255	593	145	√	GTTCAAGACACTTAGTCCTCCACAG	373	707	157	437	√
3	CAGAACCTGCTGGTGCAAGCTGGGC	1105	327	588	189	√	CAGAACCTGCTGGTGCAAGCTGGGC	1104	327	586	189	√
4	TGGGCAGGTCCTGACCAACCTGCAT	924	913	60	565	√	TGGGCAGGTCCTGACCAACCTGCAT	923	913	58	565	√
5	CACAGGTCTTCATGGGCAGGGGTTG	586	309	333	175	√	CACAGGTCTTCATGGGCAGGGGTTG	585	309	331	175	√
6	ATCCTCAGGCCTGGGCTGTAGCAAG	179	51	419	29	√	ATCCTCAGGCCTGGGCTGTAGCAAG	178	51	417	29	√
7	GCTGTCTGCCCTTGGGTTCAAGAAC	85	499	180	309	√	GCTGTCTGCCCTTGGGTTCAAGAAC	84	499	178	309	√
8	ACAAGGATACCCTCACTTTGCATGA	814	193	566	111	√	ACAAGGATACCCTCACTTTGCATGA	813	193	564	111	√
9	TTAGCCCCCACATGGGGCTGCTCTT	921	1117	380	687	√	CTGACCAGTGTCCAGTGTTAGCTCC	840	395	141	235	
10	GGCTGCTCTTGCTTCTACTAAAAGA	557	883	207	545	√	GGCTGCTCTTGCTTCTACTAAAAGA	558	883	205	545	√
11	TAAAACCAGACCCCCAGTGGATGTC	87	1045	596	645	√	TAAAACCAGACCCCCAGTGGATGTC	86	1045	594	645	√

	212054_x_at						212052 _s_at					
1	GTTCAAGACACTTAGTCCTCCACAG	372	707	158	437	√	GCTGCCTTAGACAGATTCCCTGGGC	1122	503	45	313	
2	AAGAGTGCAAGGTCTGCCAGGTCAG	668	255	592	145	√	CACCTTCCTTACACCTGGTGGGAGC	694	319	337	181	
3	CAGAACCTGCTGGTGCAAGCTGGGC	1103	327	587	189	√	TATGTGGTATGGGGGTCATTCCTGC	491	1077	179	663	
4	TGGGCAGGTCCTGACCAACCTGCAT	922	913	59	565	√	AAGTGATGGAACCCTCAGGTGCTCT	403	263	599	147	
5	CACAGGTCTTCATGGGCAGGGGTTG	587	309	332	175	√	AGCCTGAACCTCCTGACTGAGGAAC	888	143	235	81	
6	ATCCTCAGGCCTGGGCTGTAGCAAG	180	51	418	29	√	CACAGGCGTGGCTGTACACGTGCTC	157	311	554	177	
7	GCTGTCTGCCCTTGGGTTCAAGAAC	83	499	179	309	√	CTCATCATCCTTTCCAGTAACTTTA	714	379	680	221	
8	ACAAGGATACCCTCACTTTGCATGA	815	193	565	111	√	AAAAAACATCCCTCAGGTCCTGATA	916	219	212	123	
9	TTAGCCCCCACATGGGGCTGCTCTT	922	1117	379	687	√	GGTCCTGATATATTTCCTTGGATTC	1147	821	183	507	
10	GGCTGCTCTTGCTTCTACTAAAAGA	556	883	206	545	√	TTGGCTAGAAATTACACTGTGCTCA	1041	1161	505	709	
11	TAAAACCAGACCCCCAGTGGATGTC	85	1045	595	645	√	TGTGCTCAATGCCTTAATAAATCCC	842	919	38	569	

√ indicates that the probe is identical to another probe in this group of probesets.
